# Are There Common Mechanisms Between the Hutchinson–Gilford Progeria Syndrome and Natural Aging?

**DOI:** 10.3389/fgene.2019.00455

**Published:** 2019-05-15

**Authors:** Vasily V. Ashapkin, Lyudmila I. Kutueva, Svetlana Y. Kurchashova, Igor I. Kireev

**Affiliations:** Belozersky Research Institute of Physico-Chemical Biology, Lomonosov Moscow State University, Moscow, Russia

**Keywords:** aging, epigenetics, lamin, progerin, rejuvenation, reprogramming

## Abstract

The Hutchinson–Gilford progeria syndrome (HGPS) is a premature aging disease caused by mutations of the *LMNA* gene leading to increased production of a partially processed form of the nuclear fibrillar protein lamin A – progerin. Progerin acts as a dominant factor that leads to multiple morphological anomalies of cell nuclei and disturbances in heterochromatin organization, mitosis, DNA replication and repair, and gene transcription. Progerin-positive cells are present in primary fibroblast cultures obtained from the skin of normal donors at advanced ages. These cells display HGPS-like defects in nuclear morphology, decreased H3K9me3 and HP1, and increased histone H2AX phosphorylation marks of the DNA damage loci. Inhibition of progerin production in cells of aged non-HGPS donors *in vivo* increases the proliferative activity, H3K9me3, and HP1, and decreases the senescence markers p21, IGFBP3, and GADD45B to the levels of young donor cells. Thus, progerin-dependent mechanisms act in natural aging. Excessive activity of the same mechanisms may well be the cause of premature aging in HGPS. Telomere attrition is widely regarded to be one of the primary hallmarks of aging. Progerin expression in normal human fibroblasts accelerates the loss of telomeres. Changes in lamina organization may directly affect telomere attrition resulting in accelerated replicative senescence and progeroid phenotypes. The chronological aging in normal individuals and the premature aging in HGPS patients are mediated by similar changes in the activity of signaling pathways, including downregulation of DNA repair and chromatin organization, and upregulation of ERK, mTOR, GH-IGF1, MAPK, TGFβ, and mitochondrial dysfunction. Multiple epigenetic changes are common to premature aging in HGPS and natural aging. Recent studies showed that epigenetic systems could play an active role as drivers of both forms of aging. It may be suggested that these systems translate the effects of various internal and external factors into universal molecular hallmarks, largely common between natural and accelerated forms of aging. Drugs acting at both natural aging and HGPS are likely to exist. For example, vitamin D3 reduces the progerin production and alleviates most HGPS features, and also slows down epigenetic aging in overweight and obese non-HGPS individuals with suboptimal vitamin D status.

## Introduction

Progeroid (accelerated aging) syndromes are a group of monogenic diseases that resemble some aspects of natural aging. Most if not all of them are caused by mutations in DNA repair or nuclear lamina genes. These diseases could play a significant role in studies of natural aging mechanisms including epigenetic ones (Ashapkin et al., 2017). One of the most extensively studied diseases of the accelerated aging is the Hutchinson–Gilford progeria syndrome (HGPS), caused by mutations of the *LMNA* gene encoding nuclear fibrillar proteins lamins A and C (Baek et al., 2013). This syndrome is often referred to as premature aging due to multiple aging-like pathologies arising within the first few years of life. Most affected organs are those of mesenchymal origin, such as skeleton, muscle, fat, and cardiovascular system. Longevity is highly variable in HGPS patients. Most of them die at the age of 13–14 from infarction or stroke caused by progressive atherosclerosis of the coronary and cerebrovascular arteries. Progerin is a truncated version of lamin A produced via *LMNA* transcript alternative splicing. Most cases of HGPS are caused by a *de novo* 1824 C → T silent mutation in germline cells (De Sandre-Giovannoli et al., 2003; Eriksson et al., 2003), though some other mutations are known to cause syndromes with similar features.

Normal lamin A is an important constituent of the nuclear lamina, a proteinaceous network adjacent to the inner nuclear membrane and facing nuclear interior. Lamina participates in the maintenance of the nuclear structure, chromatin organization, DNA replication, and gene expression. It affects most genetic processes via attachment of the lamina-associated chromatin domains (LADs) to the nuclear periphery. The number of LADs is doubled during DNA replication. Accordingly, the expansion of lamina occurs to accommodate this increased number of LADs (Zhironkina et al., 2016).

In fibroblasts obtained from a 2-year-old HGPS patient during early passages, the nuclear morphology appears relatively normal (Goldman et al., 2004). In passage 6 cells, about 30% of nuclei display anomalous structural features (nuclear envelope lobulations), in passage 13 cells – 54%, whereas in passage 26 cells – 81%. There is also a concomitant increase in the lamina thickness. In control fibroblasts, even at passage 17, the anomalous structural features are observed in 8% of nuclei only. In fibroblasts obtained from a normal 92-year-old person, practically all nuclei look normal at passage 2, whereas about 22% of nuclei display anomalous features at passage 17. Nevertheless, these anomalous features are never as severe as those in HGPS cells. A nuclear anomaly observed in most late-passage HGPS cells only is a clustering of nuclear pore complexes in clefts formed between lobulated regions of the nuclear envelope. In control cells at all passages and HGPS cells at early passages, nuclear pores are distributed generally throughout the nuclear surface. A partial loss of the internal heterochromatin and almost a complete loss of peripheral heterochromatin occur in lobulated nuclei of the late-passage HGPS cells (Goldman et al., 2004). Instead, an unusually thick electron-dense lamina subjacent to the inner nuclear membrane appears. Accumulation of pre-lamin A concomitantly occurs in aging HGPS cells.

Transport into nuclei and incorporation into lamina occurs soon after the injection of bacterially expressed progerin into the control fibroblasts cytoplasm. This leads half an hour later to the appearance of multiple nuclei with HGPS-like abnormal morphology (Goldman et al., 2004). When a similar quantity of the normal lamin A is injected, equally rapid incorporation into nuclear lamina is observed, but the nuclear morphology remains normal. Subsequent injection of the normal lamin A does not rescue the abnormal phenotype induced by a previous progerin injection. Thus, progerin acts as a dominant factor that directly and quickly disturbs the nuclear morphology.

## Molecular Mechanisms of Progerin Production

Mature lamin A protein is produced from a precursor, pre-lamin A, via a series of post-translational processing steps: the cysteine of the C-terminal motif CAAX (C – cysteine, A – aliphatic amino acid, X – usually methionine or leucine) is farnesylated, then the terminal AAX tripeptide is proteolytically cleaved off, farnesylated C-terminal cysteine is methylated, the C-terminal 15 amino acids (647–661) are cleaved off. Both proteolytic cleavage reactions are catalyzed by a zinc metalloprotease ZMPSTE24, pre-lamin being its only known substrate. The silent C → T mutation at nucleotide position 1824 activates a cryptic donor splicing site (5′SS) in exon 11 of the LMNA transcript, thus leading to a deletion of the 3′-end proximal 150-nucleotide segment of exon 11 and production of a truncated by 50 amino acids pre-lamin A version – progerin. It does not contain 607–656 amino acid residues of pre-lamin A but does contain the C-terminal motif CAAX. Since the ZMPSTE24 protease final processing site is lost, progerin remains permanently farnesylated, leading to its stable association with the nuclear envelope. Permanent existence of the farnesylated form seems to be responsible for morphological anomalies of cell nuclei and disturbances in heterochromatin organization, mitosis, DNA replication and repair, and gene transcription.

The cryptic 5′SS in the exon 11 of the *LMNA* gene (CAG/GTGGGC) has six out of nine nucleotides of the 5′SS consensus sequence (CAG/GTAAGT), whereas in the HGPS mutant version it has seven of them (CAGGTGGGT) (Lopez-Mejia et al., 2011). This mutation is not expected to alter the strength of the 5′SS very much since it affects the terminal highly variable position (+6). Indeed, the computational analysis shows that both wild-type and mutant versions of the progerin 5′SS have much lower strength scores compared with normal lamin A 5′SS (CAG/GTGAGT) at the end of exon 11. Nevertheless, progerin is produced in HGPS mutant cells at a higher level than lamin A. The wild-type progerin 5′SS appears to be a part of a stable RNA structure that prevents its effective base pairing with U1 snRNA (Lopez-Mejia et al., 2011). The progeria mutation destabilizes this structure thus increasing the accessibility of the cryptic 5′SS to the splicing machinery. Besides, competition between the progerin and lamin A donor splice sites is more robustly regulated in favor of lamin A production by the proteins SRSF1 and SRSF6 in wild-type cells. Both these mechanisms serve to minimize progerin production during natural aging. By *in vitro* and *in cellulo* assays a sill another SR protein, SRSF5, was identified as an activator of the authentic lamin A 5′SS (Vautrot et al., 2016). Increased expression of SRSF5 in fibroblasts of HGPS patients favored the utilization of the lamin A 5′SS against the progerin 5′SS. PDGF-BB stimulation led to an increased level of SR protein phosphorylation, reduced progerin production, and partially recovered the nuclear morphology of HGPS fibroblasts.

## Mechanisms of Progerin-Induced Pathogenesis

The permanent farnesylation of progerin has been proposed to be one of the leading causes of HGPS phenotype. This suggestion was supported by a finding that inhibitors of protein farnesylation ameliorate progerin-induced anomalies. However, main HGPS phenotypes were observed in a mice HGPS model expressing a non-farnesylated version of progerin (Yang et al., 2008). The cysteine residue in the C-terminal motif CAAX of pre-lamin A has been replaced with a chemically similar serine residue, making farnesylation impossible. Nevertheless, mice expressing this progerin version developed characteristic features of HGPS (slowed growth, bone fragility, shortened lifespan, decreased subcutaneous fat), though less severe compared with mice expressing an unchanged version of progerin. These differences could be explained by the lower stability of non-farnesylated progerin molecules. Accordingly, the effects of farnesylation inhibitors could be due not to the role of farnesyl *per se*, but rather to decrease in progerin stability. On the other hand, one should take into account that the C to S replacement used prevents not only progerin farnesylation but also inhibits two subsequent steps of its processing, namely, the terminal tri-peptide cleavage and the terminal C residue methylation. Indeed, no HGPS-type anomalies were observed in mice producing a new version of non-farnesylated progerin obtained by deleting codon of the isoleucine residue in the C-terminal tetra-peptide motif (CSIM > CSM) (Yang et al., 2011).

In cultured cells, ectopic expression of the wild-type *LMNA* or progerin genes induced a progeroid phenotype – diminished replicative lifespan, senescence, and apoptosis (Candelario et al., 2008). When the wild-type *LMNA* gene is expressed, cells can be rescued by super-expression of the processing protease ZMPSTE24. Probably excessive pre-lamin A levels cause anomalies in this case. Generally, the progeroid phenotype is more severe in progerin expressing cells compared with *LMNA* expressing ones. Apparently, the lamin A metabolism is so delicately poised that even small perturbations in this system could “push” cell to a progeroid phenotype. Even in the absence of mutations, small disturbances in lamin A expression could be sufficient to cause prominent nuclear defects affecting cell aging. Probably such disturbances play a role in natural cell aging.

Cardiovascular disorders are widely believed to be among the most characteristic diseases of advanced age. Similarly, such disorders are among the most typical features of the progerin-induced accelerated aging, probably due to the accumulation of progerin in arterial walls (Olive et al., 2010). Similar to the age-associated cardiovascular pathology, there are atherosclerotic plaques and other kinds of damages (foci of calcification, inflammation, erosions) in HGPS arteries. Large quantities of progerin are accumulating in arterial walls of HGPS patients. Some progerin production also occurs in the coronary artery of normal elderly individuals and tends to increase with age. Whether this small progressive accumulation of progerin presents small chronic damage that plays a role in cardiovascular pathologies associated with natural aging is still unclear. It would be appropriate to study the progerin accumulation dynamics in non-HGPS persons with a low versus the high risk of cardiovascular pathology. It may well be that gradual accumulation of progerin, even at low levels, in vascular cells leads to an increase in cell death events and chronic inflammation response to oxidative stress and other kinds of damage. Atherosclerosis could be a consequence of these processes. Of course, this suggestion needs to be experimentally verified.

It is worth a special note that the cardiovascular aging seems to correlate not only with progerin accumulation but also with pre-lamin A accumulation (Ragnauth et al., 2010). In HGPS patients most obvious defects are observed in vascular smooth muscle cells (VSMCs). Some specific changes in these cells, such as calcification, lipid accumulation, fibrosis, and VSMC attrition, are observed both in progerin-induced and natural aging. Progressive accumulation of ultra-structural defects of nuclear lamina was observed during VSMC aging *in vivo* and in cell culture (Ragnauth et al., 2010). No accompanying progerin synthesis and accumulation was detected (RT-PCR, Western blots), but there was a significant pre-lamin A accumulation (Western blots). This accumulation of pre-lamin A occurred because of the stress-induced downregulation of the processing enzyme FACE1 (the human version of ZMPSTE24). Thus, abnormal accumulation of unprocessed pre-lamin A could induce DNA damage and mitotic dysfunction, eventually leading to cellular senescence. VSMCs are MSC-like cells that can undergo osteogenic and adipogenic differentiation, leading to calcification and lipid accumulation, pathologies prevalent in the vasculature of children with HGPS and transgenic mice overexpressing progerin. Pre-lamin A accumulation in VSMCs appears to be a causal factor of their senescence, since it precedes the senescence, and its overexpression accelerates senescence. Similar to progerin in HGPS fibroblasts, excessive pre-lamin A seems to be a dominant factor that disrupts nuclear lamina integrity, deregulates DNA damage response and mitotic checkpoints. In the context of cardiovascular disease, it could result in accelerated senescence of atherosclerotic plaques.

In both pre-lamin A-accumulating VSMCs and HGPS fibroblasts, an increase in the frequency of cells with severely fragmented nuclei immediately precedes senescence. Such nuclear damage is a characteristic feature of mitotic catastrophe, a form of cell death induced when cells carrying severe DNA damage enter mitosis. These data are in good accord with the scarcity of proliferating VSMCs and their attrition observed in advanced atherosclerotic plaques. A crucial event in the excessive pre-lamin A accumulation is oxidative stress-induced downregulation of FACE1. Oxidative damage is detectable in ∼90% of cells in atherosclerotic plaques. It may well be that such stress-induced FACE1 downregulation and pre-lamin A accumulation lead to increased DNA damage, mitotic catastrophe and premature senescence of VSMCs, and thus induce age-associated vascular pathologies.

Recently an upregulation of progerin expression was shown to occur in human dilated cardiomyopathy hearts where it strongly correlated with the left ventricular remodeling (Messner et al., 2018). Immunofluorescence study readily detected progerin-containing cardiomyocytes in dilated cardiomyopathy hearts but not in non-failing hearts. Moreover, an apoptosis marker caspase-3 and progerin were co-localized in nuclei of cardiomyocytes in dilated cardiomyopathy hearts, suggesting a possible role of progerin in apoptotic cell death. Thus, progerin may be involved in myocardial aging and eventually the heart failure.

## Does Progerin Play a Role in Natural Aging?

In control fibroblast cultures progerin could not be detected at early passages, but it appeared and accumulated at later passages (McClintock et al., 2007). In skin biopsies of variously aged (22–97-year) persons of both sexes, low levels of progerin mRNA were detected by RT-PCR using primers to exons 9 and 12 of *LMNA*, but no correlation with age was found. Small quantities of the progerin protein were detected by monoclonal antibodies in aged donor samples but not in samples from young persons. The quantity of progerin in aged donors displayed a tendency to increase with age. In primary fibroblast cultures obtained from the skin of normal donors, very few nuclei were stained with progerin-specific antibodies. The share of such nuclei was less than 0.01% in young donors and 0.3–0.8% – in aged donors. These progerin-positive cells displayed HGPS-like defects in nuclear morphology, such as nuclear envelope lobules and invaginations, binucleated cells, and others. In long-term cultures, HGPS fibroblast ceased to grow after 40–45 cumulative population doublings (CPDs), the share of the progerin-positive cells reaching about 90%. In cells obtained from normal donors in the same conditions a little increase in the progerin-positive cell share occurred. For example, in a sample obtained from an 86-year-old woman this share was about 0.4% at earlier passages and reached about 0.8% at later passages (CPDs 30–35). In total extracts of primary fibroblast cultures immunoreactive progerin was not detectable, whereas in HGPS fibroblast extracts it was readily detectable even at early passages. In extracts of a fibroblast culture obtained from an 86-year-old woman, progerin was barely detectable at later passages. Thus, progerin seems to be produced and accumulated in small quantities in wild-type fibroblasts.

In skin slices of a 9-year-old HGPS patient, progerin was detected within dermal nuclei, blood vessels, arrector pili muscle, and cells surrounding sweat glands. It was also detected in keratinocyte nuclei of the epidermis uppermost layers. In the skin of normal newborns, no progerin-positive nuclei were detected. In breast skin of a 22-year-old woman, progerin was detected in scarce cells close to the basement membrane and the papillary dermis, whereas in a 46-year-old woman, more progerin-containing cells were detected throughout the papillary dermis. Forehead skin of a 69-year-old male was found to contain progerin-positive nuclei mostly in the upper dermis, whereas a high density of progerin-positive nuclei was found throughout all layers of the dermis in the forehead skin from a 93-year-old female. Similar results were obtained with skin sections from other body sites: share of progerin-positive nuclei in the upper dermis layer was increased with age, and such nuclei emerged in deeper layers at advanced ages. Thus, a gradient of progerin-positive fibroblasts arises with natural aging between the basement membrane and the reticular dermis. Progerin-positive nuclei were not detected in most epidermis samples. The few exceptions were samples obtained from some aged donors (70–95-year) but even in these samples numbers of progerin-positive keratinocytes were by far lower compared with those of fibroblasts. Apparently, terminally differentiated or senescence cells accumulated detectable progerin. In the epidermis these cells could be keratinocytes at the end of their lifespan, immediately preceding the loss of nuclei and other organelles.

All splicing products of the *LMNA* gene were detected in primary fibroblast cultures from HGPS patients, their age-matched wild-type controls, and their unaffected parents (Rodriguez et al., 2009). Lamin C mRNA was maximally expressed in all samples (∼1500 copies per 1 μg of total RNA in HGPS and control, and ∼2000 copies in the HGPS parent fibroblasts). Lamin A mRNA concentration was practically the same in all samples and equal to that of progerin mRNA in HGPS patients (∼500 copies). Progerin mRNA levels in control and HGPS parent fibroblasts were much lower (∼2.5 copies). When early and later passages of the same cultures were compared, progerin mRNA concentration was found to be moderately increased at later passages.

Progerin mRNA and protein have been detected at low levels in human skeletal muscle samples at all ages studied (16–71-year) (Luo et al., 2013). The quantity of progerin mRNA relative to lamin A mRNA increased slightly with age, whereas progerin protein level was highly variable between individuals without apparent correlation with age. No progerin mRNA has been detected in skeletal muscle samples of variously aged mice. Thus, the splicing of *LMNA* transcripts in mice skeletal muscle cells seems to be under a more robust control.

In fibroblast cultures obtained from naturally aged human donors (81–96-year-old), nuclear aberrations similar to those in HGPS cells were consistently observed, including a decrease in H3K9me3 and HP1 levels (Scaffidi and Misteli, 2006). The frequencies of cells with such aberrations were quite comparable between naturally aged and HGPS samples, though levels of H3K9me3 and HP1 depletion were much higher in HGPS cultures. Progressive accumulation of morphological anomalies with passages was observed in fibroblast cultures obtained from normal differently aged donors. This accumulation occurred significantly earlier in cells from aged donors. The histone H2AX phosphorylation is known to mark the DNA damage loci that are still unrepaired. Numbers of such DNA damage loci were shown to be much higher in fibroblasts of both aged control individuals and HGPS patients than in fibroblasts of young donors. Thus, normal and accelerated forms of aging have much in common.

Progerin mRNA was detected by RT-PCR analysis in fibroblast cultures obtained from normal differently aged individuals. The cryptic 5′SS appeared to be used by 50-fold less frequently in wild-type compared with HGPS cells, irrespective of age. Similar results were obtained with tissue samples (liver and heart) from variously aged individuals. The quantity of immunoreactive lamin A/C does not change with age, whereas its subcellular localization changes drastically. In the young person cells, it is present both at the nuclear periphery and throughout the nucleoplasm. In the aged person cells and HGPS cells, lamin A/C is found mostly at the nuclear periphery. When the usage of progerin 5′SS was inhibited *in vivo* with a modified complementary oligonucleotide, HP1 and H3K9me3 in the aged donor cells increased to levels corresponding to the young donor cells (Scaffidi and Misteli, 2006). Moreover, expression of the senescence markers p21, IGFBP3, and GADD45B was decreased, whereas the proliferative activity was increased to the levels of young donor fibroblasts. An obvious suggestion is that progerin-dependent mechanisms play a role in natural aging, and the excessive activity of these mechanisms causes premature aging in HGPS. Age-dependent nuclear anomalies are probably caused by direct dominant action of progerin accumulated at the nuclear periphery. The continued presence of progerin even at low levels may well be one of the major causes of aging defects of nuclear structure. Aged cells could be more vulnerable to the progerin negative influence, for example, due to p53-dependent activation of the aging program.

## Molecular Mechanisms of the Progerin-Induced Aging

### Telomere Attrition

Normal human fibroblasts expressing wild-type lamin A or progerin showed reduced replicative lifespan, accelerated loss of telomeres, and morphological anomalies typical of HGPS cells (Huang et al., 2008). Probably, changes in lamina organization may directly affect the rate of telomere attrition and lead to accelerated replicative senescence and progeroid phenotypes. By quantitative fluorescent *in situ* hybridization, the average telomere length in HGPS fibroblasts was shown to be less than in normal fibroblasts (Decker et al., 2009). The telomere length of individual chromosomes is variable; none of them have consistently shorter or consistently longer telomeres. In hematopoietic cells, that do not express *LMNA*, telomeres have an equal length between HGPS patients and normal individuals. Thus, progerin expression seems to affect telomere length directly. Ectopic progerin expression in normal diploid fibroblasts was shown to activate telomere-specific DNA-damage signaling that leads to telomeres aggregation and chromosomal aberrations characteristic of telomere dysfunction (Benson et al., 2010). Vice versa, damage to telomeres in normal human fibroblasts was found to activate the progerin production and cell aging (Cao et al., 2011).

### Changed Gene Expression

In several HGPS fibroblast lines, twofold or larger changes of expression levels were observed for 361 genes (an increase for 193, and a decrease for 168) compared with control fibroblasts of similar age (Csoka et al., 2004). Genes encoding transcription factor were predominant among these differentially expressed genes: 39 totals, 29 of them known to be involved in embryogenesis and cell differentiation control. A considerable share (10 of 39) of the differentially expressed transcription factors are involved in the skeleton, limb, and muscle development. The most highly affected gene (29-fold upregulated in HGPS) was *MEOX2/GAX* that codes for a homeobox protein known to play a role in mesodermal development. Genes encoding the chromatin regulators ING1, TWIST2, and SALL1 were among the negatively affected genes. The only positively affected gene in this category was HDAC9. The second largest category contained 30 genes encoding extracellular matrix (ECM) components, such as collagens 4A1, 4A2, and 4A5, laminin α5, netrin 4, and nidogen 2. These findings are in good accord with mesodermal tissues being most severely affected in HGPS. Relative to tissue origin and function, the largest category (31 genes) was connected with the cardiovascular system and atherosclerosis. The second largest group (22 genes) was connected with skeletal system development and functioning. Transcription factors MEOX2/GAX and GATA6 super-expressed in HGPS are known repressors of VSMC proliferation.

In order to understand whether HGPS actually recapitulates natural aging or just resemble it phenotypically, cellular signaling pathways affected in natural aging *in vitro* and *in vivo* were compared with those affected in HGPS patients (Aliper et al., 2015). The data obtained showed that the natural aging and the premature aging in HGPS are mediated by similar signaling pathways, such as downregulated DNA repair, chromatin organization, caspase, and EGFR pathways, and upregulated ERK, mTOR, GH-IGF1, MAPK, TGFβ, mitochondrial dysfunction, and some other pathways.

One of the possible modes of positive co-regulation is competing for miRNA molecules between mRNAs sharing common miRNA targets (Arancio et al., 2013). The longest transcripts of the human *LMNA* gene were found to share five putative miRNA target sequences with transcripts of one gene (*DICER1*), four target sequences each – with transcripts of four genes (*CDKN1A*, *NFκB1*, *TP53*, and *VEGFA*), three target sequences – with transcripts of 12 genes, two target sequences – with transcripts of 51 genes, and one target sequence – with transcripts of 267 genes. Thus, lamin A/progerin expression appears to be highly related to the RNA interference machinery, the cell cycle regulation, and inflammation. The possible relation to epigenetic regulation of key cellular functions might partially explain many consequences of the *LMNA* gene mutations in humans.

Inducible progerin expression in a TERT-immortalized fibroblast line from human skin was shown to result in progerin accumulation at days 5–10 and some cellular defects characteristic of HGPS cells, such as aberrant nuclear morphology, increased number of DNA damage foci, and diminished lamin-associated polypeptide 2 (Scaffidi and Misteli, 2008). Super-expression of the wild-type *LMNA* gene led to similar defects, but in a much milder form. Expression of 194 genes was changed at day 5 and of 1013 genes – at day 10 after the progerin induction. About 23% of these genes were also affected by overexpression of lamin A but to a lesser degree. Several components of the Notch signaling pathway were activated early following the progerin induction. Genes encoding major direct effectors of Notch, HES1 and HEY1, were among the highest upregulated. The *HES5* gene coding for another major regulator of Notch target genes and *TLE1* coding for a transcriptional repression effector of HES proteins were also upregulated, whereas several HES target genes were downregulated. Thus, the Notch pathway activation seems to be a specific response to progerin. For all affected genes, changes in expression were already observed at 5 days after induction, whereas at 10 days these changes reached 10–15-fold levels. Expression of exogenous progerin in immortalized hMSCs induced upregulation of *HES1* and *HEY1*. Aberrant expression of cell differentiation markers was frequently observed in these MSCs, indicating spontaneous differentiation to occur. Similar but significantly lower effects were detected when lamin A was over-expressed. The effect of progerin on induced hMSCs differentiation was variable: osteogenic differentiation was stimulated, adipogenic differentiation – inhibited, chondrogenic differentiation – not affected. Similar effects were seen on the expression of a constitutively active Notch version (NICD), supporting a view of progerin action via Notch signaling pathway activation. This Notch-dependent deregulation of MSC differentiation and cell fate determination could explain multiple dysfunction phenotypes of mesenchymal tissues in HGPS. Possibly, gradual progerin accumulation with natural aging could distort the Notch pathway activity resulting in similar disturbances at a lower scale.

### The Changed Activity of Signal Pathways

Effects of progerin on levels and the sub-nuclear localization of multiple transcription regulation factors, together with changes in chromatin organization, could lead to changes in epigenetic programs in progeroid cells. A short period of hyper-proliferation in such cells is usually followed by mitotic arrest and apoptosis (Bridger and Kill, 2004). One of the possible causes of such changes may be p53 activation, but whether such activation is a direct effect of progerin or one of its secondary effects is still unclear (Liu et al., 2005). It has been noted that vitamin D receptor (VDR) knockout mice develop a premature aging phenotype that has typical features of HGPS, such as reduced lifespan, cardiovascular pathology, and some others (Bouillon et al., 2008; Keisala et al., 2009). HGPS cells were shown to have reduced expression of VDR and DNA repair factors BRCA1 and 53BP1 (Kreienkamp et al., 2016). Addition of vitamin D3 alleviated most HGPS features, such as premature senescence, inefficient DNA repair, and others. Importantly, VDR signaling affected expression of *LMNA* gene and genes of DNA repair proteins, reduced progerin production, and stabilized BRCA1 and 53BP1 proteins in HGPS cells. Obviously, progerin could impact the VDR gene epigenetic status, leading to its repression, thus contributing to HGPS phenotype. Transfection of retinal pigment epithelial cells with progerin caused replication fork stalling and nuclease-mediated degradation (Kreienkamp et al., 2018). This probably leads to cytoplasmic accumulation of single-strand DNA fragments and induction of a cell-intrinsic IFN-like response. Indeed, about half-hundred genes in IFN/antiviral/innate immunity pathway were found to be upregulated in HGPS and downregulated by vitamin D3.

The multiplicity of signaling pathways affected in HGPS and their interdependence makes it difficult to distinguish direct targets of progerin from secondary affected ones. NFκB was shown to play an important role in the regulation of gene expression in mammalian aging (Adler et al., 2007). In HGPS mice models, accumulation of pre-lamin A or progerin at the nuclear periphery was shown to activate the NFκB signaling pathway, thus contributing to the senescence-associated secretory phenotype (SASP) (Osorio et al., 2012). Such NFκB activation could give rise to systemic inflammation inherent in aging (inflammaging) (Franceschi and Campisi, 2014). NFκB may be a major driver of accelerated aging since its inhibition significantly increases lifespan in HGPS model mice (Osorio et al., 2012).

The transcription factor NRF2 is widely known to be related to longevity (Lewis et al., 2015). It activates the expression of antioxidant genes via binding to antioxidant-responsive promoter elements (ARE). NRF2 has also been identified as a primary target of progerin that drives its aging-acceleration effects (Kubben et al., 2016). More than 200 of putative NRF2 target genes were found to be significantly downregulated in HGPS fibroblasts compared with their wild-type counterparts. Similar to progerin, NRF2 was preferentially accumulated near the nuclear envelope in HGPS cells. Progerin displayed enhanced binding to NRF2 compared with lamin A. Thus, it may impair the activity of NRF2-ARE antioxidant regulation pathway through sequestration of NRF2 away from its target genes.

Deacetylase proteins of the sirtuin family SIRT1 and SIRT6 are known to play important roles in mammalian aging (Ashapkin et al., 2017, 2018, and references therein). By pull-down immunoprecipitation assays SIRT1 has been shown to interact with lamin A but not with pre-lamin A or progerin (Liu et al., 2012). Moreover, the specific deacetylase activity of SIRT1 toward p53 was increased in the presence of lamin A in a dose-dependent manner. Resveratrol has been reported to increase SIRT1 activity toward the fluorophore-conjugated synthetic p53 peptide but not its native form (Borra et al., 2005; Kaeberlein et al., 2005; Pacholec et al., 2010). Surprisingly, in the presence of lamin A, resveratrol did increase the SIRT1 deacetylase activity toward its native target, acetyl p53, by enhancing the association between SIRT1 and lamin A (Liu et al., 2012). A significant part of total SIRT1 has been detected in the nuclear matrix fraction in wild-type cells, whereas dissociation of SIRT1 from this fraction was observed in cells ectopically expressing pre-lamin A or progerin. Thus, progerin seems to compromise the proper localization of SIRT1. In the *Zmpste24^-/-^* mouse model of HGPS, resveratrol supplementation alleviated progeroid features and increased lifespan. Interestingly, lamin A was shown to physically interact with another aging-related sirtuin – SIRT6 (Ghosh et al., 2015). This interaction enhances the histone H3 deacetylating activity of SIRT6 toward H3K9ac and H3K56ac. Progerin also binds to SIRT6 but does not affect its H3K9ac- and H3K56ac-specific activity. SIRT6 is known to play an important role in DNA repair, being recruited to DNA loci with double-strand breaks, where it increases the activity of PARP1 via its mono-ADP-ribosylation (Mao et al., 2011). Both recruitment of SIRT6 and PARP1 activation appeared to depend on lamin A and to be inhibited by progerin (Ghosh et al., 2015). Probably these effects of progerin are responsible for a significant part of the genome instability in HGPS.

### Disturbance of Chromatin Organization

A progressive loss of H3K27me3-dependent X chromosome inactivation (Xi) with passages was observed in HGPS fibroblast cultures from a female patient but not in age-matched control fibroblast cultures (Shumaker et al., 2006). The H3K27me3 loss seemed to be the earliest event followed by loss of Xi and appearance of anomalies in nuclear structure. A significant loss of H3K27me3-specific histone methylase *EZH2* mRNA was also detected in HGPS fibroblasts but not in their normal counterparts. No loss of Xi-associated *XIST* RNA was observed in HGPS and control fibroblasts. In a wild-type human cell line, transient progerin expression led to a rapid loss of H3K27me3 associated with Xi and nuclear lamina but no loss of Xi-associated *XIST* RNA was detected. Thus, expression of endogenous progerin in HGPS cells and of exogenous progerin in normal cells leads to a similar loss of H3K27me3 epigenetic marks from facultative heterochromatin probably explaining its decondensation. A similar loss of another heterochromatin-specific histone methylation mark H3K9me3 was observed in HGPS cells at later passages as well as in wild-type cells expressing exogenous progerin (Shumaker et al., 2006). In wild-type cells and early passage HGPS fibroblasts, H3K9me3 marks are mostly co-localized with heterochromatin protein HP1α. In HGPS cells at later passages, loss of both H3K9me3 and HP1α and reduction of expression of genes encoding H3K9me3-specific histone methylases SUV39H1 and SUV39H2 occur. Unexpectedly, another heterochromatin marker, H4K20me3, was found to be upregulated both in HGPS fibroblasts and in wild-type cells expressing exogenous progerin.

In control human skin fibroblasts, wide patches of H3K27me3 were found at gene-poor regions devoid of CpG islands (CGIs), whereas more localized H3K27me3 enrichment was observed at specific CGI containing promoters (McCord et al., 2013). A decrease or even loss of H3K27me3 large patches in gene-poor regions was often observed in HGPS fibroblasts compared with normal cells. This feature was significant at the genome-wide scale and correlated with a lower level of mRNA encoding the major H3K27me3-specific methyltransferase EZH2. Besides these broad changes, local increases or decreases of H3K27me3 level were observed at specific CGI containing promoters. Globally among the genes that changed expression at least fourfold between HGPS and control fibroblasts, downregulated genes preferentially gained H3K27me3, whereas upregulated genes preferentially lost H3K27me3, consistent with the widely known inhibitory effect of H3K27me3 on gene expression. A correlation was observed between loss of H3K27me3 wide patches in gene-poor regions and loss of their association with lamin A/C, preferentially at the nuclear periphery. It was suggested that chromatin enriched in H3K27me3 is physically associated with the nuclear lamina. This could explain the increasing loss of peripheral heterochromatin in HGPS cells. In normal cells, gene-poor inactive regions of chromatin tend to be near the nuclear periphery, while active, gene-rich regions are in the nuclear interior. Hi-C analysis of late-passage HGPS fibroblasts showed an almost complete loss of spatial separation between open (active) and closed (inactive) chromatin compartments. A global loss of chromosome compartments seems to occur catastrophically throughout the genome when HGPS cells approach a prematurely senescent stage. Some compartment changes were also observed in normal senescent cells, but these changes were never as drastic as in HGPS cells. Besides, certain genomic regions changed their compartment identity in HGPS compared with normal cells, and these changes correlated with the changes in H3K27me3 and lamin A/C association. Regions that changed from the open compartment in normal cells to a closed compartment in HGPS cells were found to gain H3K27me3 and lamin binding, while those changed from closed to open compartment lost H3K27me3 and lamin binding. The data described are in good accord with the popular view of the progressive breakdown of repressive chromatin structure that initiates spurious transcriptional events and promotes aging via loss of inhibiting epigenetic marks (Ashapkin et al., 2018).

One of the mechanisms that could explain the disturbance of chromatin structure and the accelerated senescence of HGPS cells is the changed localization of the histone H3K4me3 reader proteins of the ING family. These proteins are known to facilitate DNA repair through quick changes in gene expression as part of cellular responses to genotoxic insults (Soliman and Riabowol, 2007). It has been shown that lamin A specifically binds ING1 protein thus playing an essential role in its nuclear targeting (Han et al., 2008). Progerin does not bind ING1. These notions could explain the changed subcellular distribution of ING1 in HGPS cells. ING1 is known to play a role in active H3K4me3-directed DNA demethylation (Schäfer et al., 2013). Thus, progerin-induced changes in ING1 distribution could globally affect DNA methylation. Indeed, impaired DNA demethylation in *Gadd45a/Ing1* double-knockout mice has been shown to lead to a segmental progeria phenotype (Schäfer et al., 2018).

### Changed DNA Methylation

DNA methylation changes are widely believed to be a major driver of natural aging and may be essential in accelerated aging. Multiple differences in methylated loci were found between cells of progeroid patients and their control counterparts (Heyn et al., 2013). There was a common part of differentially methylated loci in Werner syndrome (WS) and HGPS, but most of them were specific for one syndrome. Atypical WS and HGPS patients that had none of the known *WRN* or *LMNA* gene mutations displayed similar changes of DNA methylation though lesser compared with mutant patients. Individual variability in the extent of DNA methylation changes was rather high between *WRN* mutant patients. Methylation of 144 CpGs was found to be consistently changed both during natural aging and in WS. Respective genes could be involved in natural and accelerated aging. In samples of atypical WS caused by *LMNA* gene mutations, 18480 differentially methylated CpGs were identified, of which 485 were variably methylated during natural aging. The locus *LOC149837* encoding a long non-coding RNA (lncRNA) with unknown function and a group of piRNAs was differently methylated in all progeria samples. An obvious suggestion is that its anomalous hypomethylation could change the expression of these ncRNAs, thus contributing to the progeroid phenotype.

Of particular note, the use of the pan-tissue DNA methylation clock revealed epigenetic age acceleration in segmental progeroid syndromes such as Down syndrome and typical WS, but not in HGPS and atypical WS patients (Horvath et al., 2018). By contrast, the application of the novel skin and blood DNA methylation clock showed that fibroblasts from HGPS patients exhibited accelerated epigenetic aging, especially evident in HGPS children who are younger than 10 years old. A non-significant trend of increased epigenetic age was found in atypical WS cases caused by low levels of progerin.

As was noted above (see section “The Changed Activity of Signal Pathways”) addition of vitamin D3 to HGPS cells reduces the progerin production and alleviates most HGPS features. In a randomized clinical study, vitamin D3 supplementation decreased DNA methylation age in overweight and obese individuals with suboptimal vitamin D status (Chen et al., 2019). Interestingly, the correlation between DNAm age and chronological age was higher at the beginning of the study than post-test, suggesting that the vitamin D3 supplementation was driving the epigenetic age to deviate from the chronological age. Thus, the activity of vitamin D signal pathway appears to counteract both premature aging in HGPS and natural aging.

### Are There Common Epigenetic Mechanisms Between Natural and Accelerated Aging?

Epigenetic reprogramming of somatic cells could be regarded as an example of complete age resetting (Ashapkin et al., 2017, 2018, and references therein). It has been shown that iPSCs produced from somatic cells of centenarian donors are quite similar to embryo stem cells (ESCs) by most molecular age markers, such as gene expression profile, telomere length, and others. Moreover, similar to ESCs, such iPSCs have an epigenetic age close to zero and could be differentiated into somatic fully rejuvenated cells. The reprogramming efficiency is known to decline with aging due to the accumulation of senescence features that prevent efficient reprogramming (Li et al., 2009; Kim et al., 2010). The efficiency of reprogramming of HGPS fibroblasts at passages 15–20 was fourfold lower compared with control fibroblasts (Zhang et al., 2011). Nevertheless, all individual iPSC colonies were morphologically indistinguishable from ESCs. Human HGPS fibroblasts-derived iPSCs did not express progerin and lacked disease-specific features. However, when these iPSCs were differentiated into somatic cells of mesenchymal lineages, such as fibroblasts, VSMCs, or MSCs, progerin re-expression was observed, and these cells displayed increased DNA damage and nuclear abnormalities. A genome-wide study of CpG methylation in HGPS fibroblasts, control fibroblasts, HGPS-iPSCs, control iPSCs, and control human ESCs showed that generated iPSC lines were much closer to each other and ESCs than the two fibroblast lines (Liu et al., 2011). The presence of progerin in HGPS fibroblasts appeared to lead to major epigenetic changes that disappeared in HGPS-iPSCs concomitant with the downregulation of progerin. Genome-wide mRNA profiling also showed that HGPS-iPSCs and control iPSCs were closely related to each other and ESCs, and quite different from their parental fibroblasts. These data demonstrated the complete resetting of the epigenome and the gene expression profile in HGPS cells after being reprogrammed to pluripotency. The HGPS model *LmnaG609G* mice exhibit the accelerated onset of many age-associated features in multiple organs and shortened lifespan (Osorio et al., 2011). Short-term inducible expression of the Yamanaka factors (Oct4, Sox2, Klf4, and c-Myc – OSKM) for 2 or 4 days in fibroblasts obtained from such mice did not lead to pluripotency but significantly improved the nuclear envelope architecture and reduced the foci of histone γH2AX, a known marker of DNA damage associated with aging (Ocampo et al., 2016). Furthermore, downregulated expression of age-related stress response genes in the p53 pathway (*p16^INK4a^*, *p21^CIP1^*, *Atf3*, and *Gadd45B*) and the senescence-associated *MMP13* and *IL6* genes was observed. SA-β-gal activity and the production of mitochondrial ROS were also reduced. Last but not least, levels of two heterochromatin-specific epigenetic marks, H3K9me3, and H4K20me3 that change with aging were restored. Importantly, restoration of H3K9me3 occurred earlier than the reduction of γH2AX foci and improvement of nuclear architecture. Thus, epigenetic remodeling could be an active driver of the aging phenotype amelioration. Since the *in vivo* induction of OSKM is known to lead to the teratoma formation and the cancer development, a safe cyclic OSKM induction protocol was elaborated. Interestingly, cyclic OSKM induction during 6 weeks in *LmnaG609G* mice did not alter lamin A/C or progerin expression but ameliorated multiple features of aging at molecular, cell, tissue, and organismal levels and extended lifespan. Similar to *LmnaG609G* cells, in late-passage wild-type cells, short-term induction of OSKM diminished histone γ-H2AX foci, downregulated the expression of age-related stress response genes in the p53 pathway and senescence-associated *MMP13* and *IL6* genes, reduced the production of mitochondrial ROS, and restored levels of H3K9me3. Collectively these results suggest that cyclic *in vivo* OSKM induction may retard the aging by preventing epigenetic changes, increase in cellular senescence pathway activity, and other molecular hallmarks of aging. Thus, cell rejuvenation without changing cell identity is principally possible. A different possibility of changing cell identity without rejuvenation has been demonstrated by induced direct conversion of human fibroblasts into neurons (Mertens et al., 2015). Unlike neurons obtained via intermediate iPSCs, directly induced neurons retained the epigenetic signature of the donor age. Interestingly, of more than 200 genes differentially expressed between neurons obtained from young and old donors, only seven overlapped between fibroblasts and neurons, whereas 49 overlapped between induced neurons and human prefrontal cortex samples. In aging-related expression profiles, only three genes were shared between fibroblasts, induced neurons, and the brain, namely *LAMA3*, *PCDH10*, and *RANBP17*. Since the fibroblast-specific epigenetic signature of donor age was somehow transformed into the neuron-specific one, a logical suggestion would be that a putative master regulator of aging was among these shared genes. The nuclear pore-associated transport receptor *RANBP17* gene was significantly downregulated with age, indicating a possible functional impairment of the nuclear pore function. Indeed, induced neurons from the oldest donors showed significant nucleocytoplasmic compartmentalization defects compared with middle-aged and young donor-derived neurons, and the extent of these defects correlated with donor age and *RANBP17* downregulation. Furthermore, an shRNA-mediated downregulation of *RANBP17* in young fibroblasts changed the expression of most fibroblast aging genes in the same direction, as was observed at natural aging. Thus, downregulation of *RANBP17* expression may be a causative factor in cellular aging. As expected, iPSCs-derived neurons showed essentially rejuvenated transcriptomes and no detectable impairment of nucleocytoplasmic compartmentalization. The accelerated aging phenotype in HGPS is a consequence of progerin-impaired nuclear envelope structure that might lead to nuclear leakiness and disruption of nucleocytoplasmic compartmentalization. Thus, a decrease of *RANBP17* expression in aging cells can contribute to their aging-related phenotypes. It is widely believed that iPSCs have a limitless ability to be propagated and re-differentiated into fully rejuvenated somatic cells. However, it was shown that prolonged iPSCs cultivation leads to metabolic changes that prevent their neuronal differentiation (Masotti et al., 2014). Young iPSCs do not express the *LMNA* gene, whereas aged iPSCs were found to express high *LMNA* mRNA levels and have a thickened nuclear lamina containing large amounts of lamin A/C, very much like HGPS and senescent cells (Petrini et al., 2017). These features of aged iPSCs were associated with nuclear anomalies resembling those of HGPS cells. Moreover, increased expression of pre-lamin A and progerin was observed in aged iPSCs, as well as hyperactivation of NFκB and downregulation of SIRT7 expression, known to regulate mitochondrial biogenesis. Thus, upon prolonged propagation, iPSCs accumulate age-related changes common with normal aging and progeroid syndromes.

Homozygous WRN-null lines of hESC expressed pluripotency markers and were able to differentiate into all three germ layer cells (Zhang et al., 2015). WRN-null MSCs that were derived from WRN-null ESCs expressed MSC-specific cell surface markers and could be differentiated into osteoblasts, chondrocytes, and adipocytes. These WRN-deficient MSCs displayed main features of accelerated aging, such as reduced proliferative potential, increased SA-β-gal markers, upregulated expression of senescent marker genes *p16^*Ink4a*^* and *p21^*Waf1*^*, and activation of SASP. DNA damage markers 53BP1, γH2AX, and phosphorylated ATM/ATR substrates were increased. Moreover, these cells showed downregulation of heterochromatin-associated proteins LAP2b and LBR of the inner nuclear membrane and diminished peripheral heterochromatin. Significant downregulation of the constitutive heterochromatin mark H3K9me3 was observed, whereas H3K27me3 showed only slight downregulation. No significant changes in 5mC and euchromatin mark H3K4me3 were found. About 70 H3K9me3-enriched “mountains” ( >20 kb of consecutive peaks of H3K9me3) were identified in wild-type MSCs, 28 (38%) of them being absent in WRN-deficient MSCs. Most of these lost H3K9me3 mountains were located in sub-telomeric or sub-centromeric regions. RNA sequencing identified 1047 genes that showed differential expression between WRN-deficient and wild-type MSCs. The most obviously downregulated genes were those encoding centromere-packaging proteins and components of the nuclear membrane. Immunoprecipitation analysis showed WRN to be a part of the complex containing H3K9me3 specific methyltransferase SUV39H1, heterochromatin-associated protein HP1α, and lamin-associated protein LAP2b, suggesting WRN to be involved in heterochromatin stabilization. Comparison of the heterochromatin mark levels between wild-type MSCs derived from young (7- to 26-year) and old (58- to 72-year) individuals showed significant downregulation of WRN protein and H3K9me3, HP1α, SUV39H1, and LAP2b marks in MSCs derived from old individuals. Thus, specific heterochromatin changes underlie both accelerated and natural MSC aging.

## Conclusion

Even small disturbances in lamin A expression could be sufficient to cause nuclear defects affecting cell aging. Probably such disturbances play a role in natural cell aging. Small numbers of progerin-positive cells are present in primary fibroblast cultures obtained from the skin of normal donors at advanced ages. These cells display HGPS-like defects in nuclear morphology, such as nuclear envelope lobules and invaginations, binucleated cells, and decreased levels of heterochromatin marks H3K9me3 and HP1. The histone H2AX phosphorylation mark of the DNA damage foci is significantly increased in fibroblasts of elderly individuals and HGPS patients compared with young donors. In the young person cells, lamin A/C is present both at the nuclear periphery and throughout the nucleoplasm, whereas in the aged person and HGPS cells it is found mostly at the nuclear periphery. Inhibition of progerin production in cells of aged non-HGPS donors *in vivo* increases their proliferative activity, H3K9me3, and HP1 levels, and decreases the expression of senescence markers p21, IGFBP3, and GADD45B to the levels of young donor cells. Thus, progerin-dependent mechanisms act in natural aging. The excessive activity of these mechanisms in HGPS may be the main cause of premature aging. Telomere attrition is widely regarded to be one of the primary hallmarks of aging. Normal human fibroblasts expressing progerin show accelerated loss of telomeres. Probably, changes in lamina organization may directly affect the rate of telomere attrition and lead to accelerated replicative senescence and progeroid phenotypes. Interestingly, damage to telomeres in normal human fibroblasts activates the progerin production and cell senescence, suggesting the existence of a vicious circle. The chronological aging in normal individuals and the premature aging in HGPS patients are mediated by similar changes in the activity of signaling pathways, including downregulation of DNA repair and chromatin organization, and upregulation of ERK, mTOR, GH-IGF1, MAPK, TGFβ, and mitochondrial dysfunction.

Lamin A/progerin expression appears to be highly related to the RNA interference machinery, DNA and histone methylation, and chromatin structure. Relation to epigenetic regulation of key cellular functions might explain many consequences of the *LMNA* gene mutations in humans. The Notch-dependent deregulation of MSC differentiation and cell fate determination could explain multiple dysfunction phenotypes of mesenchymal tissues in HGPS. Gradual progerin accumulation with the natural aging could distort the Notch pathway activity resulting in similar disturbances in a milder form. Accumulation of progerin at the nuclear periphery activates the NFκB signaling pathway, possibly contributing to systemic inflammation inherent in aging (inflammaging). NFκB may be a major driver of accelerated aging since its inhibition significantly increases lifespan in HGPS model mice.

The longevity-related transcription factor NRF2 is one of the primary targets of progerin that drive its aging-acceleration effects. More than 200 of NRF2 target genes are significantly downregulated in HGPS. Via its increased affinity to NRF2 progerin may impair the activity of NRF2-ARE antioxidant regulation pathway through sequestration of NRF2 away from its target genes.

Progerin seems to compromise the proper localization of the sirtuin family members SIRT1 and SIRT6 known to play important roles in mammalian aging. Probably these effects are responsible for a significant part of the genome instability in HGPS.

A global loss of chromosome compartments occurs catastrophically throughout the genome when HGPS cells approach a prematurely senescent stage. Similar changes occur in normal senescent cells, though these changes are never as drastic as in HGPS cells. These data are in good accord with the popular view that a progressive breakdown of repressive chromatin structure takes place in naturally aged cells and initiates spurious transcriptional events via loss of inhibiting epigenetic marks. ING1 plays a role in active H3K4me3-directed DNA demethylation. Progerin changes its distribution and thus may globally affect DNA methylation. Indeed, impaired DNA demethylation in *Gadd45a/Ing1* double-knockout mice has been shown to lead to a segmental progeria phenotype.

**FIGURE 1:**
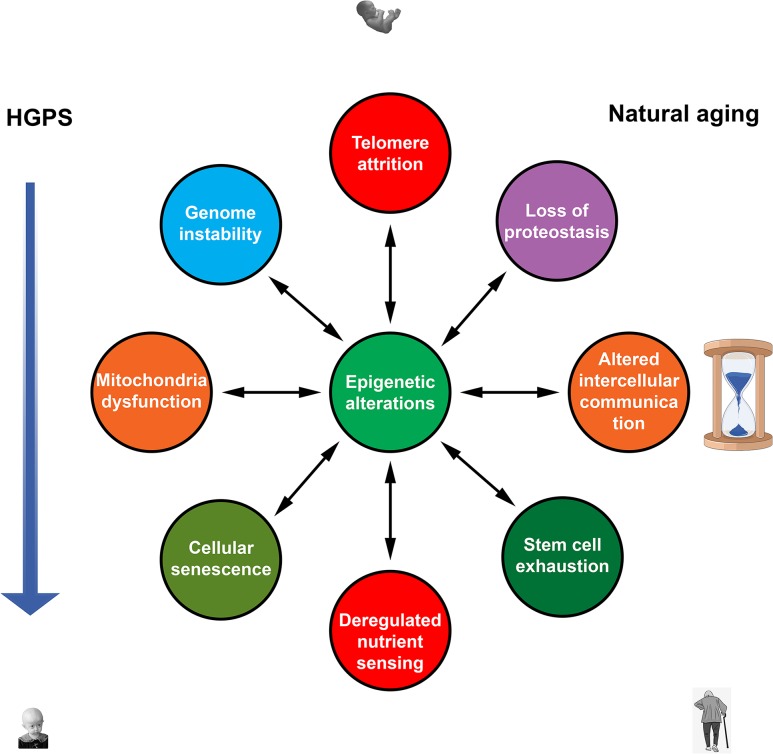
Nine hallmarks of aging (Lopez-Otın et al., 2013) contribute to both natural and premature aging. Epigenetic alterations are in a key position to affect all hallmarks of aging and to be affected by them. Mutual influences between other hallmarks that probably exist are not shown.

DNA methylation changes are widely believed to be a primary hallmark of natural aging and may be essential in premature aging (Figure 1; Lopez-Otın et al., 2013). DNA methylation clocks revealed an aging acceleration in HGPS and other segmental progeroid syndromes. Vitamin D3 reduces progerin production and alleviates most HGPS features. It also decreases epigenetic age in overweight and obese individuals with suboptimal vitamin D status. Thus, the activity of vitamin D signal pathway appears to counteract both premature aging in HGPS and natural aging.

Cyclic *in vivo* OSKM induction may retard the accelerated and natural aging by counteracting epigenetic changes, increased cellular senescence, and other molecular hallmarks of aging.

The nuclear pore-associated transport receptor *RANBP17* gene is significantly downregulated with age, indicating a possible functional impairment of the nuclear pore function and significant nucleocytoplasmic compartmentalization defects. The accelerated aging phenotype in HGPS is a consequence of progerin-impaired nuclear envelope structure that might lead to nuclear leakiness and disruption of nucleocytoplasmic compartmentalization.

Young iPSCs do not express the *LMNA* gene, whereas aged iPSCs were found to express high *LMNA* mRNA levels and have a thickened nuclear lamina containing large amounts of lamin A/C, very much like HGPS and senescent cells. These features of aged iPSCs were associated with nuclear anomalies resembling those of HGPS cells. Moreover, increased expression of pre-lamin A and progerin was observed in aged iPSCs, as well as hyperactivation of NFκB and downregulation of SIRT7 expression, known to regulate mitochondrial biogenesis. Thus, upon prolonged propagation, iPSCs accumulate age-related changes common in natural aging and progeroid syndromes.

Cardiovascular disorders are among the most typical features of both natural aging and HGPS. Large quantities of progerin are accumulating in arterial walls of HGPS patients. Increased progerin production with aging also occurs in the coronary artery of normal individuals. Specific changes in VSMCs, such as calcification, lipid accumulation, fibrosis, and VSMC attrition, are observed both in progerin-induced and natural aging.

**FIGURE 2:**
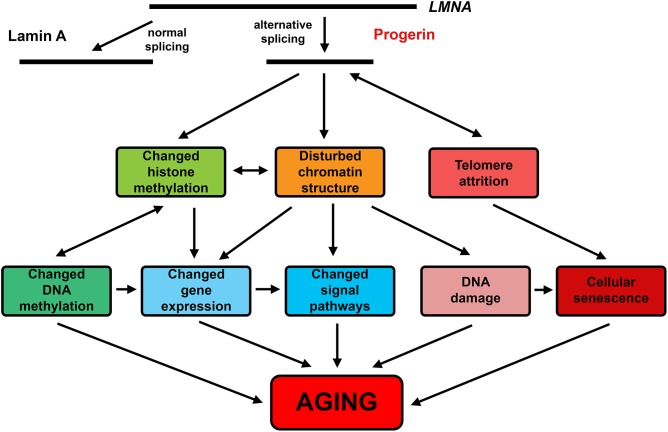
The effects of progerin common in HGPS and natural aging.

Collectively the data described above show that most features of natural aging are found in HGPS. While the primary cause of HGPS is quite different, the secondary downstream causes and their consequences at the organismal level are very similar to natural aging (Figure 2). Thus, HGPS indeed represents bona fide accelerated aging, not merely a semblance of aging.

Despite all that is known about HGPS and the progerin action, many questions remain to be answered (Gonzalo et al., 2017). One of the most enigmatic features of HGPS is that some organs, for example, liver and brain, appear to be quite healthy. Some characteristics typically observed in normal aging, such as progressive intellectual disability, are absent in HGPS. In progerin-expressing HGPS-like mice, accelerated aging is observed, but brain function seems to be mostly unaffected (Yang et al., 2005; Jung et al., 2012). One of the possible explanations is a very low level of the *LMNA* gene expression in brain cells due to the post-transcriptional downregulation by a brain-specific miRNA, miR-9 (Jung et al., 2012). Lamin A is not produced in induced pluripotent stem cells (iPSCs), obtained from fibroblasts of HGPS patients and control subjects (Nissan et al., 2012). Induction of *LMNA* expression occurs in many iPSC-derived cells, such as keratinocytes, MSCs, and some others. In cells derived from HGPS-iPSCs typical consequences of the progerin expression were observed, such as accelerated senescence, chromatin derangement, and disturbances in nuclear morphology. No expression of the *LMNA* gene was observed in iPSC-derived neurons in good accord with miR-9 expression. Exogenous miR-9 downregulated lamin A and progerin production and alleviated anomalies in nuclear morphology in HGPS MSCs. Thus, brain cells seem to be protected from progerin-induced damage in HGPS patients by a brain-specific miRNA. Forced expression of human progerin in murine hippocampal neurons led to HGPS-like anomalies in nuclear structure (Baek et al., 2015). Nevertheless, the chromatin structure and gene expression remained unaffected (only 5 genes of 16572 investigated showed twofold or larger changes in expression levels), and no changes in brain functioning were detected. Obviously, there are some other means of brain resistance to progerin, besides its low expression.

Given the widely known association between cancer and aging, a quite surprising finding was that progeroid patients do not develop cancer. It has been shown that progerin may inhibit cell transformation by changing the distribution of the general transcriptional regulator BRD4 on chromatin and activating tumor-protective cellular pathways (Fernandez et al., 2014).

Another intriguing question is why the progerin cryptic splice site exists at all? Due to the degeneracy of the genetic code, the respective sequence of *LMNA* exon 11 could lose any similarity to the consensus 5′SS without changes of the encoded amino acid sequence. Nevertheless, the splice site responsible for progerin production appears to be highly conserved throughout placental mammals (Lopez-Mejia et al., 2014). One of the possible explanations could be that progerin existence has some evolutionary advantage. Indeed *Lmna* knock-in mice in which progerin-specific splicing cannot occur showed increased lifespan but, somewhat paradoxically, decreased energy metabolism, increased weight gain and fewer mitochondria, whereas HGPS-like mice had a short lifespan but increased mitochondrial biogenesis (Lopez-Mejia et al., 2014). Moreover, old progerin-free mice showed dramatically increased frequency of lymphoid tumors in the abdominal cavity compared with wild-type mice. Thus, progerin seems to be involved both in tumor protection and metabolic adaptations.

Hopefully, as the research proceeds, the answer to these questions will be found, and the new mysteries of aging will be revealed. Anyhow, HGPS offers a unique model for elucidating the roles of progerin in the cell aging.

Multiple epigenetic changes are common to premature aging in HGPS and chronological aging in normal individuals. Recent studies showed that epigenetic systems could play an active role as drivers of both forms of aging. An ability of the epigenetic systems to affect all other drivers of aging via changes in gene expression puts them in a unique position. It may be suggested that these systems translate the effects of various internal and external factors into universal molecular hallmarks, mostly common between natural and accelerated forms of aging (Figure 1). Unlike other kinds of damage, age-related epigenetic changes could be fully or partially reversed to a “young” state. Furthermore, such epigenetic remodeling to a more youthful state could largely ameliorate effects of other aging denominators. Hence, targeted interventions in epigenetic mechanisms appear to be the most promising and effective strategy for developing therapies to combat both natural and accelerated aging.

## Author Contributions

All authors listed have made a substantial, direct and intellectual contribution to the work, and approved it for publication.

## Conflict of Interest Statement

The authors declare that the research was conducted in the absence of any commercial or financial relationships that could be construed as a potential conflict of interest.
